# Supplementary material for: Engaging narratives evoke similar neural activity and lead to similar time perception
Source: Sci Rep. 2017 Jul 4;7:4578. doi: 10.1038/s41598-017-04402-4 (PMC5496904; doi:10.1038/s41598-017-04402-4)
Supplement: Supplementary file 1 — Supplementary Information [file 41598_2017_4402_MOESM1_ESM.pdf]

SUPPLEMENTARY INFORMATION

Engaging narratives evoke similar neural activity and lead to similar time perception

Samantha Cohen, Simon Henin, and Lucas C. Parra

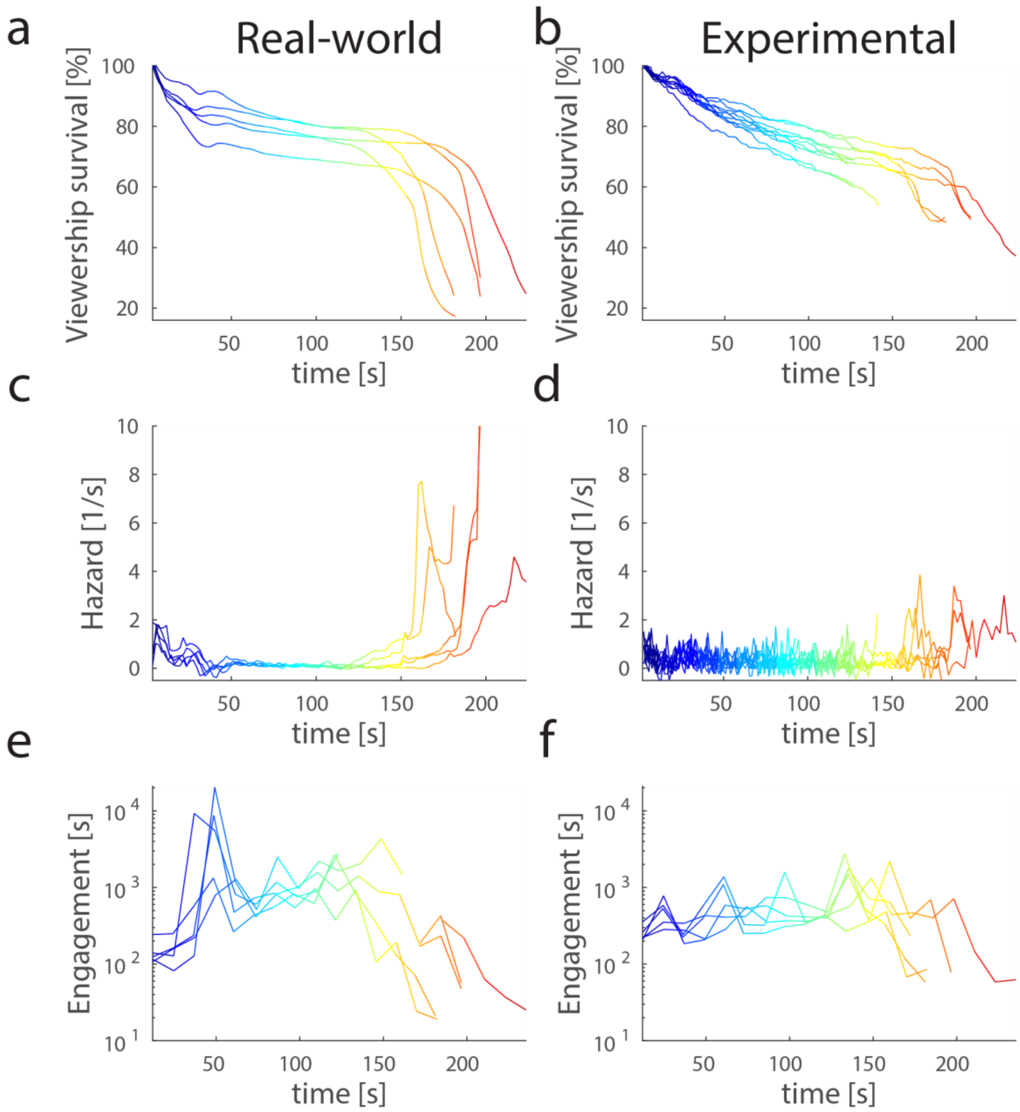

Supplementary Figure S1: Viewership survival translates into behavioral engagement in both "real-world" and "experimental" cohorts. Engagement data collected in the real-world (online viewers, watching at their discretion, 5 videos, left column), and experimentally (viewers recruited on MTurk, 10 videos, middle column). **a/b:** Survival curves, S(t), show percent of viewers retained as a function of stimulus time. **c/d:** Hazard (Equation 3) or risk of viewer loss over time. Note that the hazard for the Experimental cohort is more noisy as compared to real-world cohort. This is to be expected as this is a much smaller sample of viewers (in a 12 s interval viewership drops in average by 9000 in the real-world cohort, but only by 7 in the smaller experimental cohort). **e/f:** Engagement (Equation 1) for the five videos that were the same for the real-world and experimental cohorts.

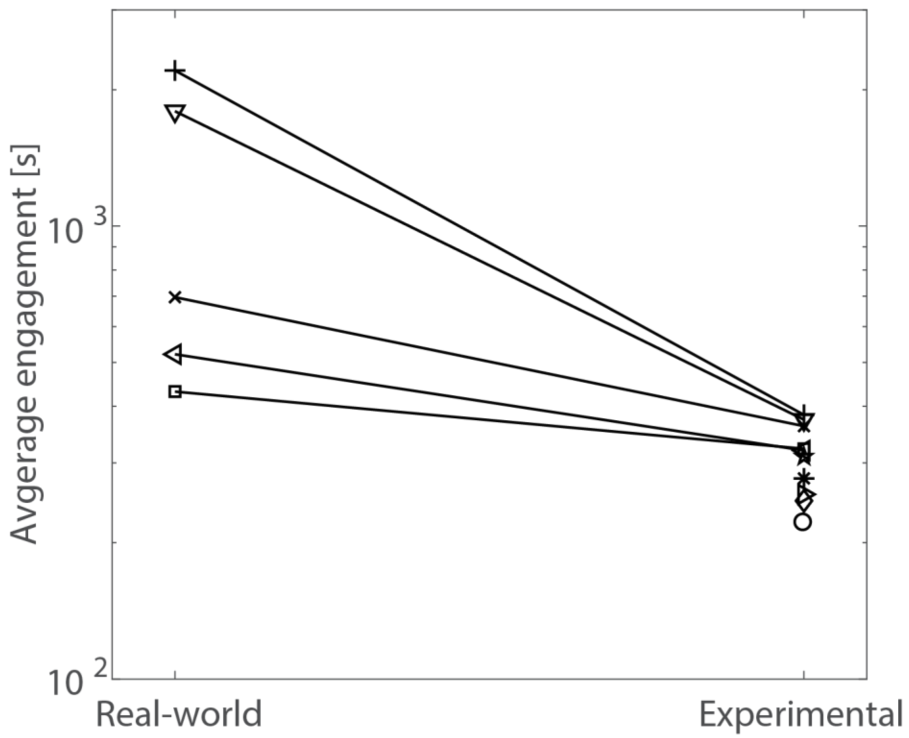

Supplementary Figure S2: Real-world Engagement is higher than the engagement measured experimentally. Lines connect the videos common to both the “Real-world” cohort, where viewership accrued organically, and “Experimental” cohort, where viewership was directed via Amazon’s Mechanical Turk (MTurk) platform. Real-world viewers were significantly more engaged than those recruited experimentally ($t(4)=3.2$, $p=0.03$, paired t-test). The 5 additional videos acquired for the Experimental cohort are also indicated as individual points.

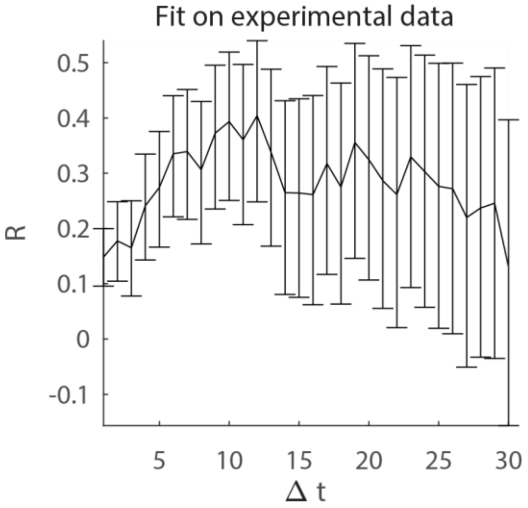

Supplementary Figure S3: Goodness of fit (R) between the experimental behavioral engagement, $E(t)$, and neural engagement, $E_0\, \gamma(\boldsymbol{t})$, calculated for different time intervals Δt. Δt=12s is selected for all analyses because it is a good compromise between performance and number of samples (smallest p-value, R(12s)=0.4, p=2e-6). Error bars represent the 95% confidence interval for each R value.

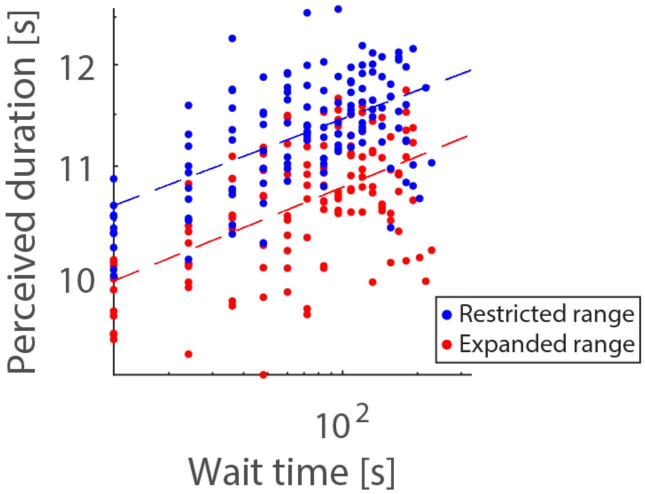

Supplementary Figure S4: Perceived time duration depends on wait time. Viewers estimated the duration of time intervals within each video (N = 129 time intervals). Each point represents a time interval in a video. When viewers had to wait longer for the interval of interest (because it was later in the video), the duration of the interval was also perceived as lasting longer (r = 0.57, p = 2e-12, and r = 0.54, p = 2e-11, N = 129, for restricted and expanded range, respectively). Comparisons are made for two independent cohorts which had either a restricted range (blue, 8-16s) or expanded range (red, 4-20s) available for their time duration estimates. All time measures are displayed on a perceptual log-seconds scale.

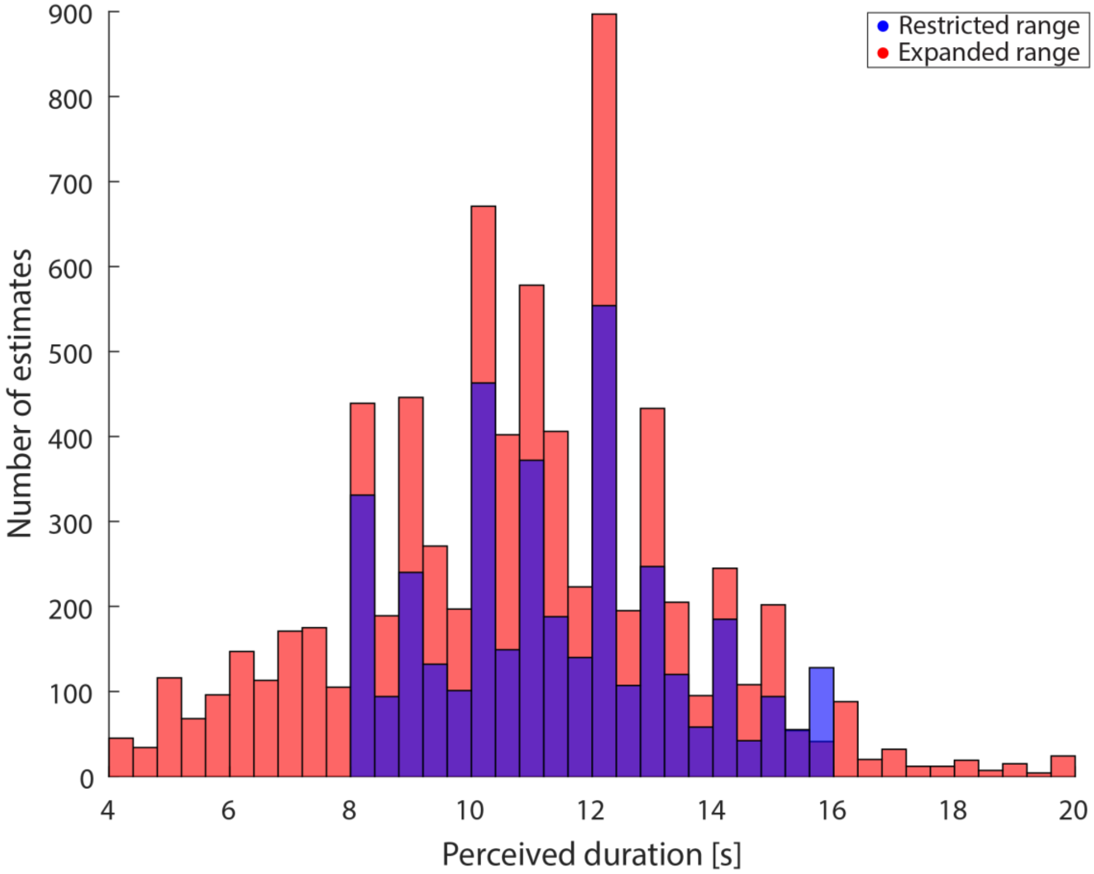

Supplementary Figure S5: Distribution of perceived time duration for both experimental cohorts. The distribution of perceived time estimates appears to be truncated in the restricted range cohort who estimated values between 8 and 16 seconds (380 subjects, blue) in comparison to the expanded range cohort who estimated values between 4 and 20 seconds (720 subjects, red).

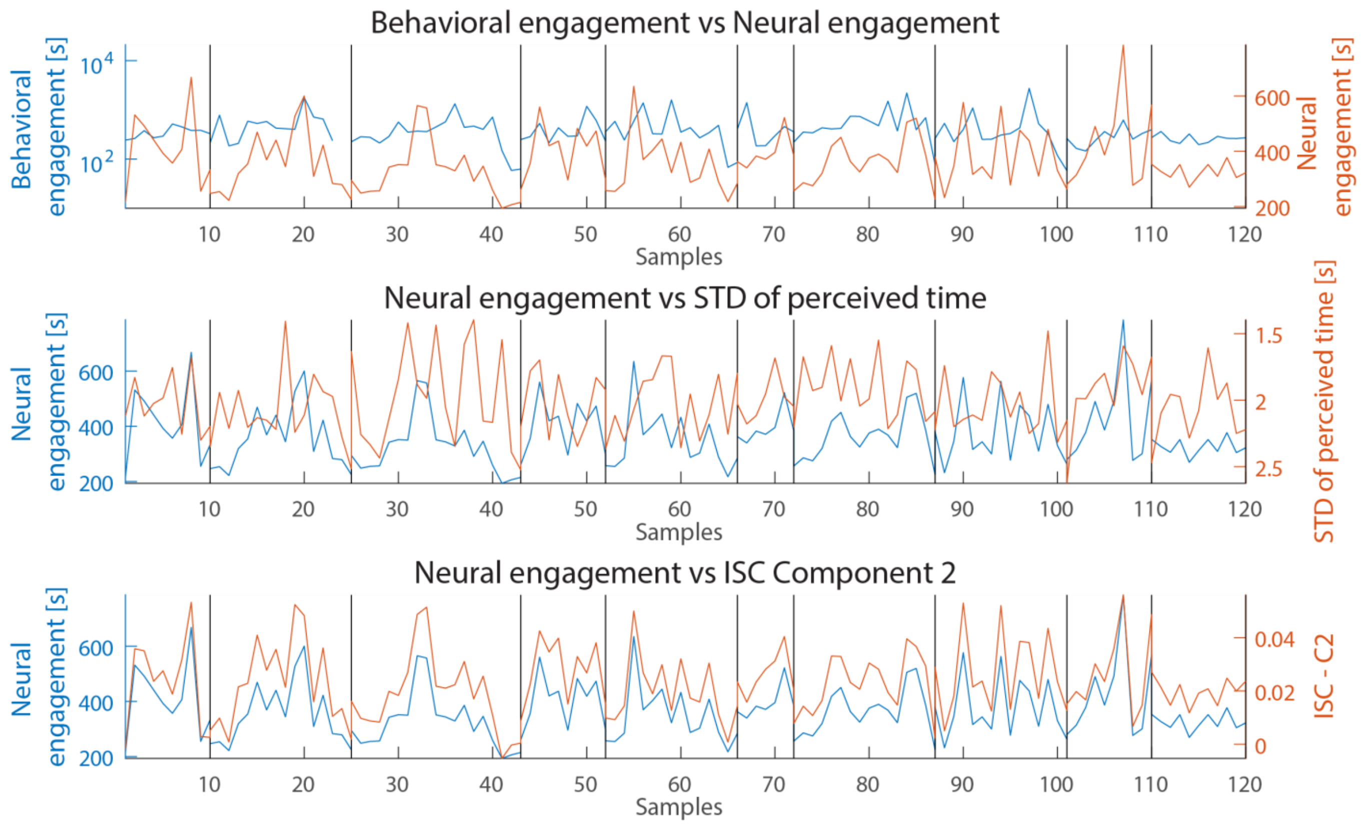

Supplementary Figure S6: Time courses for scatterplots reported in text. a: Experimental behavioral engagement (blue) compared with neural engagement (orange). **b:** Neural engagement (blue) compared with the standard deviation of time estimates across viewers (both cohorts pooled, orange). **c.** Neural engagement (blue) compared with the second component of inter-subject correlation (ISC – C2, orange). This component contributes most strongly to neural engagement. Vertical lines divide time courses for each video. Time is presented in samples of 12s duration.
